# Facebook Advertising for Disseminating Early Psychosis Education to the Chinese-Speaking General Public

**DOI:** 10.1089/heq.2018.0087

**Published:** 2019-02-13

**Authors:** Benjamin K.P. Woo

**Affiliations:** Asian Pacific Health Corps, University of California, Los Angeles, Los Angeles, California.

**Keywords:** Chinese, Facebook, first-episode psychosis, health education, schizophrenia, YouTube

## Abstract

**Purpose:** To explore the role of Facebook advertisement in disseminating first-episode psychosis knowledge to the Chinese-speaking general public.

**Methods:** A Facebook advertisement, linked to an external YouTube education video on first-episode psychosis, was displayed for 48 h. Metrics such as number of unique individuals reached, the number of engagements, the engagement rate (engagements/reaches), and the cost per post engagement were recorded. Data were analyzed using descriptive statistics.

**Results:** Facebook advertisement reached a total of 252 unique viewers and prompted 66 post engagements.

**Conclusion:** Facebook advertisement was able to target younger Chinese-speaking individuals at risk of developing early psychosis.

## Introduction

Schizophrenia is a major public health problem worldwide. Typically, psychosis begins in late adolescence and eventually interferes with social functioning. Approximately 2.5% of Chinese adult population had lifetime psychotic disorder.^1^ Early intervention in psychosis, as well as shortening duration of untreated psychosis, may improve long-term recovery rate of patients with schizophrenia.^2^ Previous studies have shown that Chinese populations fail to recognize the risks and signs of early psychosis.^3,4^ Therefore, assessment of cost-effective ways to provide information to the general public about first-episode psychosis is an important step toward schizophrenia treatment and prevention in Chinese communities.

The Internet has proven to be an important source to disseminate health knowledge to Chinese-speaking populations.^5–8^ Not only is the Internet a primary source for information gathering for patients and their families during first-episode psychosis, it is also an important channel to raise general public awareness about prevention and early intervention of psychosis. Previous studies have demonstrated that YouTube videos were successful in disseminating mental health and first-episode psychosis knowledge to Chinese populations.^9–11^ However, few studies have investigated the role of Facebook advertisement in promoting Chinese-language YouTube health videos in ethnic health outreach. This study aims to examine the usefulness of Facebook advertisement at sharing a YouTube video on first-episode psychosis knowledge with Chinese-speaking populations.

## Methods

A Facebook advertisement was on display for 48 h and consisted of a 6-character Chinese title and a 26-character text body. The advertisement invited the viewers to click the ad to watch an external informational video on YouTube about first-episode psychosis education taught in Chinese (https://youtu.be/qbPmEiqA8Ho). The video covered the etiology, treatment, and methods of prevention of early psychosis.

Because first-episode psychosis typically begins in late adolescence or early adulthood, we chose to target those of ages 18–34 years with our advertisement. This study includes Facebook users who could read traditional Chinese and saw the Facebook ad during the 48-h advertisement period. Institutional Review Board approval was obtained through the University of California, Los Angeles.

Data were obtained by Facebook Analytics and YouTube Analytics. Demographic information including age and gender were recorded. Parameters included the number of individuals reached, the number of impressions, the number of engagements, the engagement rate (engagements/reaches), and the cost per post engagement. Data obtained through Facebook analytics were further confirmed using data obtained directly from the video link through YouTube analytics during the 48-h campaign.

## Results

The 48-h campaign reached a total of 252 unique viewers, comprising 145 (57.5%) female and 107 (42.5%) male viewers. All 252 (100%) unique viewers accessed the ad through mobile devices. Furthermore, 148 (57.1%) women and 107 (42.9%) men generated a total of 259 impressions. The calculated cost per 1000 impressions was $25.17 for this Facebook campaign.

The Facebook advertisement prompted a total of 66 post engagements. These engagements included those who clicked on the advertisement link, those who like the advertisement, and those who shared the advertisement link. The overall engagement rate was 26.2%. The advertisement generated 38 engagements from 145 female viewers (engagement rate of 26.2%). Conversely, the advertisement generated 28 engagements from 107 male viewers (engagement rate of 26.2%). The cost per post engagement among female viewers was $0.10, whereas the cost per post engagement among male viewers was $0.09.

In the 18–24 years age group, the advertisement reached 143 (56.7%) viewers with 40 post engagements (engagement rate of 28.0%). In the 25–34 years age group, the advertisement reached 109 (43.3%) viewers with 26 post engagements (engagement rate of 23.9%). The cost per post engagement was $0.10 for both groups.

During the 48-h advertisement run, the video had 13 views on YouTube. There was a total watch time of 92 min, and the average view duration was 7.10 min (28.8% of total video length). In comparison, the lifetime average view duration of this video was 5.48 min (22.2% of total video length).

## Discussion

To our knowledge, this study is the first to report on the effect of Facebook advertising to engage young Chinese-speaking adults and enhance their knowledge on first-episode psychosis and schizophrenia. Online recruitment methods have been used for conducting surveys, but few studies have reported on their use for disseminating health knowledge to the general public.^12^ The main findings were that Facebook advertisement is a useful channel to target younger Chinese-speaking individuals to obtain information on first-episode psychosis, especially the 18–24 years age group. Also, viewers directed from the Facebook advertisement were more interested in early psychosis education, as evidenced by the longer average view duration for the YouTube education video on first-episode psychosis.

A previous study indicated that Chinese-speaking individuals were likely to believe that patients with schizophrenia are dangerous and uncontrollable.^13^ Public stigma and discrimination can have devastating effects on Chinese with first-episode psychosis. Nevertheless, a meta-analysis found that education had positive effects on reducing public stigma for adults with a mental illness.^14^ Also, studies have shown that early detection and intervention in people with psychosis can be achieved with widespread informational campaigns and easily accessible services.^15,16^ Our study demonstrates that Facebook advertisement was able to attract Chinese-speaking viewers to online mental health education resources at low cost, as well as correctly target the advertisement at the desired age group at risk of developing psychosis. Future studies should compare Facebook advertisement with traditional recruitment methods, such as pamphlets or mail, and monitor how samples of young adults recruited through ads seek out further psychoeducation.^17^

In addition, 100% of the advertisements were viewed through mobile devices. Internet access on mobile phones is commonplace, and future studies should explore how to effectively utilize m-health as a method to disseminate psychoeducation. Moreover, although Facebook is the most popular social media avenue among young adults in the United States, other popular social media networks including Twitter, Snapchat, and Instagram are on the rise. E-mental health or m-health can be widely distributed worldwide and be readily available for prevention and psychoeducational efforts on first-episode psychosis.^7,10^ Therefore, it will be interesting in the future for studies to assess the effectiveness of recruiting younger participants for psychoeducation through other social media networks.

A limitation of this study is the lack of demographic information on the viewers such as ethnicity and socioeconomic status. Furthermore, as the Facebook advertisement and YouTube video were fully conducted in Chinese, the study is restricted to those who can read and understand Chinese. In addition, the advertisement was only run for 48-h and longer trending would provide more data. Finally, the study was not able to assess a participant's reason for clicking the advertisement or evaluate knowledge on first-episode psychosis before and after viewing the video.

## Conclusion

Timely education for the general public on first-episode psychosis remains a significant public health challenge. Overall, our data showed that utilizing social media to advertise and promote psychoeducation among young adults can be a cost-effective alternative to traditional means. Further research efforts should be targeted at understanding, developing, and implementing m-health as a way to disseminate psychoeducation to improve mental health knowledge for the Chinese-speaking communities.
